# Thiazolidinedione induced thyroid associated orbitopathy

**DOI:** 10.1186/1471-2415-7-8

**Published:** 2007-04-26

**Authors:** Seongmu Lee, Angelo Tsirbas, Robert A Goldberg, John D McCann

**Affiliations:** 1 Division of Orbital and Ophthalmic Plastic and Reconstructive Surgery, Jules Stein Eye Institute, and the Department of Ophthalmology, David Geffen School of Medicine at UCLA, Los Angeles, California, USA; 2 Center for Facial Appearances, Salt Lake City, Utah, USA

## Abstract

**Background:**

To describe an episode of thyroid associated orbitopathy (TAO) following the initiation of thiazolidinedione (TZD).

**Case presentation:**

We report a female patient with a history of Graves' disease and stabilised thyroid associated orbitopathy for 2.5 years, who experienced rapid progression of TAO after the initiation of thiazolidinedione for glycemic control. Following the discontinuation of TZD, the patient experienced subsequent stabilisation of disease and normalization of vision. The medical history, ophthalmic findings, and clinical course are discussed.

**Conclusion:**

Thiazolidinediones may exacerbate TAO, and this should be taken into consideration when selecting treatment for diabetic patients with a history of autoimmune thyroid disorders.

## Background

The thiazolidinediones (TZDs) are among one of several classes of oral hypoglycemic agents commonly utilized to maintain glycemic control in patients with type 2 diabetes mellitus. While the mechanism by which TZDs increase the action of insulin is not precisely known, these agents have been shown to be potent agonists of the nuclear hormone receptor, peroxisome proliferator activated receptor-γ (PPAR-γ), which is found predominantly in adipose tissue and plays a dominant role in adipocyte differentiation [1]. A primary cause of proptosis in thyroid associated orbitopathy is the expansion of adipose tissue volume in the orbit. We report a case of a female patient with a history of Graves' disease and stabilized TAO, who experienced rapid progression of proptosis following initiation of rosiglitazone for glycemic control. This study was performed in accordance with the Declaration of Helsinski.

## Case presentation

In February 2005, a 56-year-old female smoker presented to our clinic for follow-up of her Graves' disease. Eyelid retraction was noted two years prior to presentation, but she reported no proptosis or other signs of TAO. She was diagnosed with hyperthyroidism in August 2004 and had been stabilized on propranolol and propylthiouracil. In November 2004, the patient was placed on rosiglitazone/metformin for glycemic control. Over the next several months thereafter, the patient reported development of diplopia on upgaze and rapid progression of proptosis, particularly in the left eye. She also noticed an increase in abdominal girth and a greater amount of subcutaneous fat in her abdomen, chest, and back.

**Figure 1:**
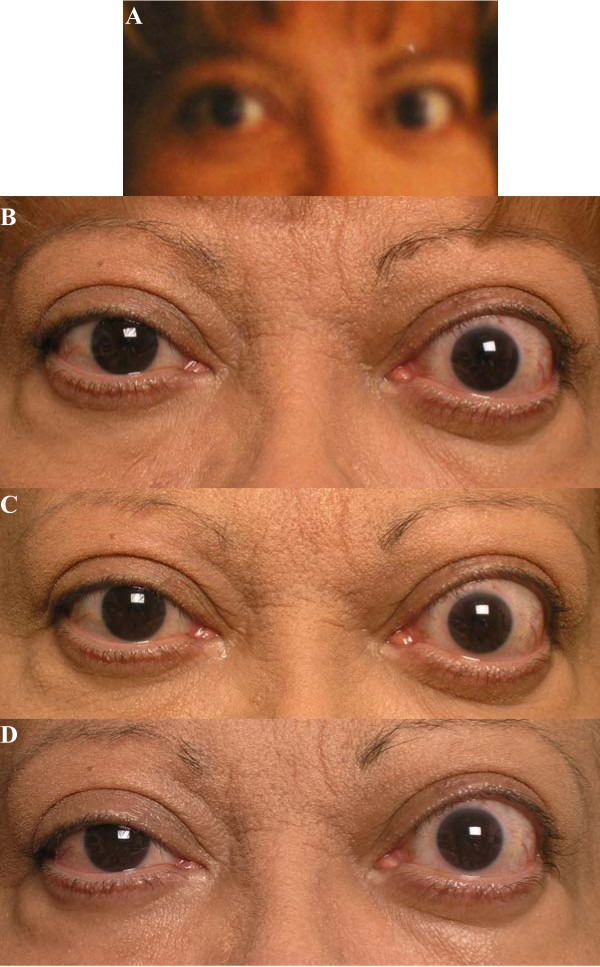
A. Patient, previous photograph prior to initiation of TZD. B. Patient, on initial visit, with evidence of eyelid retraction and severe proptosis. C. Patient, at six-week follow-up visit. D. Patient, at 3 month follow-up visit.

On clinical examination we noted evidence of eyelid retraction and severe proptosis, with Hertel measurements of 25 mm on the right and 28 mm on the left (Figure 1B). Extraocular motility was within normal limits, and visual acuity without correction was 20/40 in both eyes. Intraocular pressure was within normal limits, and there were no signs of exposure keratopathy. There was evidence of a subtle optic neuropathy on the left with mild red color desaturation and an afferent pupillary defect. Thyroid function tests showed a euthyroid state. An elevation of TBII was noted, but other thyroid autoantibody screens were negative.

Treatment with rosiglitazone/metformin was discontinued, and the patient was started on metformin and glipizide to maintain glycemic control. There had been consideration by her medical team to utilize I131 to radioactively ablate the thyroid gland. However, given the possibility of transiently worsening her thyroid-related orbitopathy, the decision was made to pursue medical management with propranolol and propylthiouracil.

**Figure 2:**
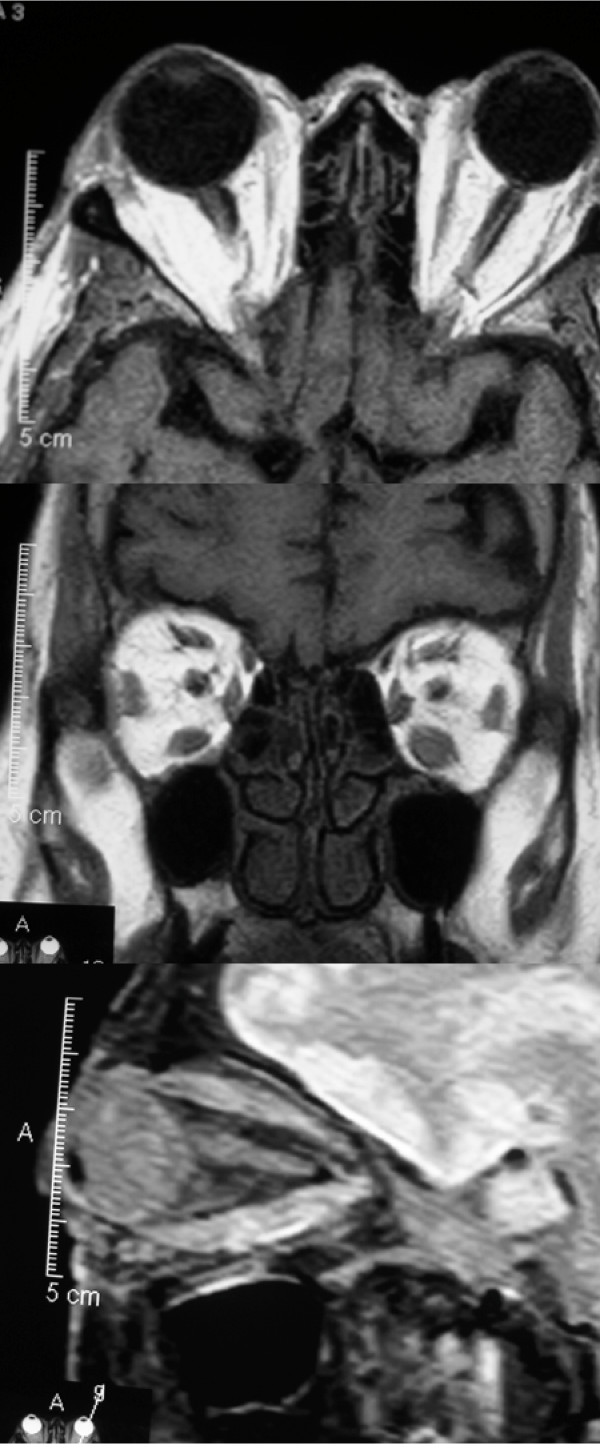
Magnetic resonance imaging scans of patient demonstrating expansion of the orbital fat compartment and enlargement of the left inferior rectus muscle.

On 6-week follow-up, there was no progression of disease (Figure 1C). Hertel measurements were 26 mm on the right and 29 mm on the left, and her vision remained stable, with resolution of the pupillary defect. MRI of the orbits performed in April 2005 revealed expansion of the orbital fat compartment and mild enlargement of the inferior rectus muscles on each side (Figure 2). Of note, the patient was found to have a right thyroid nodule, which, on cytological examination of samples obtained by fine-needle aspiration, revealed atypical cells. She proceeded to have a right hemithyroidectomy for further management. Microscopic analysis of surgical specimen revealed papillary carcinoma, predominantly follicular variant, which had been completely excised.

Following partial thyroidectomy, early synthroid replacement was initiated to prevent hypothyroidism and possible deterioration of her TAO. Based on her MRI findings and profound proptosis on the left side, the decision was made to proceed with orbital decompression surgery prior to further treatment of the papillary cancer with I131 ablation. Physical examination findings were unchanged, with Hertel measurements 25 mm on the right and 29 mm on the left (Figure 1D).

## Discussion

TAO is a common cause of proptosis [2]. Exacerbation of TAO, however, is uncommon once the stable phase of the condition is reached, and causes of flare ups are not well understood but may include I131 treatment, hypothyroidism, and smoking [3-5].

A well-known side effect of TZDs is an increase in subcutaneous fat [1]. Recent studies have suggested a link between the expression of the thyroid stimulating hormone receptor (TSHr) and adipogenesis in the orbital tissues of patients with TAO [6,7]. Specifically, the activation of PPAR-γ by its agonist, TZD, was shown to stimulate functional TSHr expression, and also to induce the recruitment and differentiation of orbital fibroblasts into mature lipid-laden adipocytes [7-9]. This potential for preadipocyte differentiation is shared with abdominal subcutaneous tissue. Other studies have shown that the expression of PPAR-γ was greater in adipose and connective tissue from patients during the active stages of TAO [10,11]. These results suggest that TSHr expression in orbital fibroblasts may be linked to adipogenesis, and that the activation of the PPAR-γ, such as by TZD, may play an important role in the stimulation of adipogenesis and the pathogenesis of TAO.

These findings, in addition to the temporal profile of this patient's TAO exacerbation, the development of TAO about 24 months after her initial dysthyroid state whilst euthyroid, and the subsequent stabilisation of proptosis following discontinuation of the rosiglitazone, suggest that the TZD may have had a role in the pathogenesis of this patient's proptosis. Similar cases of thyroid eye disease exacerbation following the initiation of TZD have been reported in the literature [12,13], and a recent study by Dorkhan et al. showed that a subgroup of patients with type 2 diabetes mellitus treated with pioglitazone responded with increased eye protrusion [14]. Although TAO can occur this late after the initial systemic diagnosis, we believe the temporal association, in addition to the patient's other body habitus changes, strongly suggest a role of rosiglitazone in this case. These studies also suggest that potential therapies for TAO may be directed towards inhibition of the adipogenic pathway through the use of a PPAR-γ inhibitor, although the detrimental metabolic effects should be considered, and that PPAR-γ expression could potentially be utilized as a marker for TAO disease activity [7,8].

## Conclusion

TZDs are a commonly utilized hypoglycemic agent in the treatment of diabetes mellitus, and their precise roles in fat metabolism continue to be elucidated. As agonists of the PPAR-γ, its effects may have important implications in the selection of treatment modalities for patients with diabetes, and it will be important to be aware of potential problems in patients with a history of autoimmune thyroid disorders, even in those without obvious evidence of TAO.

## Competing interests

The author(s) declare that they have no competing interests.

## Authors' contributions

SL wrote the initial draft and assisted in its final preparation and submission. AS was involved in the care of the patient and helped write the manuscript. RAG helped in the management of the case. JDM was the primary surgeon involved in the care of the patient. All authors have read and approve the final manuscript.

## Pre-publication history

The pre-publication history for this paper can be accessed here:


